# Effects of stochastic resonance whole-body vibration on sensorimotor function in elderly individuals—A systematic review

**DOI:** 10.3389/fspor.2023.1083617

**Published:** 2023-04-17

**Authors:** Slavko Rogan, Jan Taeymans

**Affiliations:** ^1^ Department of Health, Discipline of Physiotherapy, Bern University of Applied Sciences, Bern, Switzerland; ^2^ Faculty of Physical Education and Physiotherapy, Free University of Brussels, Brussels, Belgium

**Keywords:** stochastic resonance therapy, whole-body vibration (WBV), falls, aged, postural balance [MeSH]

## Abstract

**Introduction:**

Due to demographic changes, falls are increasingly becoming a focus of health care. It is known that within six months after a fall, two thirds of fallers will fall again. Therefore, therapeutic procedures to improve balance that are simple and can be performed in a short time are needed. Stochastic resonance whole-body vibration (SR-WBV) may be such a procedure.

**Method:**

An electronic search to assess the effectiveness of SR-WBV on balance in the elderly was conducted using databases that included CINAHL Cochrane, PEDro, and PubMed. Included studies were assessed using the Collaboration Risk of Bias Tool by two independent reviewers.

**Results:**

Nine studies showing moderate methodological quality were included. Treatment parameters were heterogeneous. Vibration frequency ranged from 1 to 12 Hz. Six studies found statistically significant improvements of balance from baseline to post measurement after SR-WBV interventions. One article found clinical relevance of the improvement in total time of the “Expanded Time to Get Up and Go Test”.

**Discussion:**

Physiological adaptations after balance training are specific and may explain some of the observed heterogeneity. Two out of nine studies assessed reactive balance and both indicated statistically significant improvements after SR-WBV. Therefore, SR-WBV represents a reactive balance training.

## Introduction

Ageing is associated with sensorimotor deficits resulting in muscular weakness, mobility issues, balance disorders and in gait disorders, and this leads to falls and reduced independence in everyday life (1). The sensorimotor system constantly and unconsciously regulates its movements and postural control based on perceived information to achieve postural stability. Numerous research studies have assessed the effect of stochastic resonance (SR) stimulation to the lower extremity on postural regulation and balance performance in sub-populations such as healthy adults (mean age: 23.04 years, ±6.33 years) (2), elderly individuals (mean age: 73.00 years) (3), or individuals with comprised health suffering from Parkinson (4), or multiple sclerosis (5) or stroke (6, 7), SR has been shown in a variety of physiological systems (8–11), in which the presence of noise below the sensory threshold could enhance the response of the system to weak signals (12–14). Collins et al. (12) postulated that SR could be used to elderly individuals with elevated sensory thresholds.

Whole body vibration (WBV) with stochastic resonance (SR) can easily applied. SR-WBV does not lead to exhaustion and blood pressure and lactate levels are low during vibration training (15). SR-WBV could be easily personalized to the individual's level of fitness (16). For example, elderly with low baseline fitness who want to start an exercise program should start with a program that meets their physical capabilities (16). Older people with frailty or pre-frail condition are advised to undergo a “skilling-up” phase before undertaking more traditional forms of training (17). SR-WBV can be used as a training modality for the “skilling-up” phase (16, 18, 19). Compared to traditional balance training, there are indications on how to design a training regime (sets, rest between sets, session per weeks, etc.) and on the other hand the training protocols are characterized by a short duration between 1 and 5 min of intermittent or continuous WBV application (20). There is no need to change clothes or shoes or to shower afterwards, which might be important in the working world or for adults who do not want to waste time on intensive training (2). Eichelberger et al. (21) were able to determine a decrease in accelerations with increasing distance from the vibrating plate due to damping properties of the involved body structures. However, it is known that a prolonged exposure to vibration (e.g., driving, hammering) may lead to musculoskeletal and neurological disorders (22, 23). Systematic reviews and meta-analysis (24–27) have shown that shorter exposure to vibration have a positive effect on muscle strength and postural control if the training regime (e.g., amplitude, duration and frequency of vibration) is correctly dosed.

SR-WBV differs from sinusoidal WBV in that the stimuli are randomized and amplified using noise (25, 28). This results in a generation of action potentials by the suprathreshold stimuli (29). SR-WBV induces an excitatory stimulus to the alpha motoneuron *via* mono- and polysynaptic pathways and elicits muscle activation in response, resulting in body stabilization (30). SR-WBV can be understood as reactive balance training that simulates a fall situation itself through the application of unpredictable, random, and multidirectional displacements of the stance surface (31). Reactive balance training means that a person has the ability to react to a loss of balance, because reactive balance is a key factor that ultimately determines whether an individual will sustain a fall (32). Reactive balance can be profoundly impaired in older adult populations (32).

In contrast to SR-WBV, sinusoidal WBV are constant. If the stimulus remains the same, the body adapts very quickly and this slows down the impact of growth stimulus (27). Three WBV devices were used in clinical settings: sinusoidal vertical (SV-WBV), sinusoidal side-alternating (SS-WBV), and stochastic resonance (SR-WBV). While the sinusoidal WBV devices uses a single plate for standing, the SR-WBV device uses two plates for standing (24, 33–36). Due to the different physiological mechanisms of impact and use of equipment, this paper focuses on SR-WBV.

Furthermore, study results demonstrated that whole-body vibration training provides more than physiological effects (2, 37). Animals study showed that daily exposure to WBV over five weeks significantly improved cognition in young mice compared to non-vibrated mice (38, 39). Regterschot et al. (37) could determine that passive WBV could improve executive functions in healthy young adults. They postulated that WBV has the potential as a cognition-enhancing therapy. Chan et al. (40) reported that executive functions are a set of cognitive processes that regulate, manage and control other cognitive processes in order to achieve a goal, such as planning, mental flexibility, multi-tasking etc. Research findings described that cognitive decline and falls are linked (41–43) and that cognitive training improve balance and gait (44).

### Aim

A systematic literature review on the effects of SR-WBV on postural control have been conducted previously (27). As the number of publications on SR-WBV has increased significantly in recent years, this present systematic literature review aims to provide an update on the status quo of the efficacy of SR-WBV on postural control in frail elderly individuals. The research question was: could SR-WBV positively influence postural control in individuals with balance disability?

## Methods

### Study design

This paper is an update of the systematic review by Rogan et al. (27). In advance, a registration on PROSPERO (CRD420203194) was conducted and the guideline “Preferred Reporting Items for Systematic Reviews and Meta-Analyses” (PRISMA) was used for reporting. This current systematic review used the same methodological approach as the first study. The inclusion and exclusion criteria were identical. The same search terms were used on the same databases. The data collection process was more comprehensive in this study. Besides the training load, the intervention protocol and the measurement instrument tools were now included. The risk of bias was assessed with the same instrument (The Cochrane Collaboration Cochrane Risk of Bias Tool) as in the first study.

#### Information sources

Electronic searches were conducted on CINAHL, Cochrane Central Register of Controlled Trials, Physiotherapy Evidence Database (PEDro), and PubMed up to August 2022. In addition, a hand search of the reference lists of included studies, research institution websites, and Google Scholar was conducted.

### Search strategy

The PICO model was used in this study. The PICO acronym stands for **P**opulation (elderly, frail elderly), **I**ntervention (WBV exercise), **C**omparator (no treatment, or other balance exercise), **O**utcomes (postural control, static, dynamic, functional balance). Search terms included: (i) “stochastic resonance whole-body vibration” OR “SR-WBV” OR “stochastic vibration” OR “stochastic training” AND (ii) “balance” OR “postural control* “ OR “postural stability”.

#### Eligibility criteria

This study included intervention studies and randomized controlled pilot studies. German- and English-language articles with intervention and control groups from the fields of geriatrics were considered. For studies with frail elderly persons, those aged 65 years and older were eligible. Studies with frail elderly persons under 65 years of age, studies with elderly persons with “fit” status, and studies with neurological diseases were excluded.

#### Data collection process

Two independent study nurses screened and analyzed the title and abstract for inclusion and exclusion criteria. In the next step, the full text was read and included in this systematic literature review if eligible. For each included article, authors, population, intervention protocol, outcome parameters, results, and training load were extracted and electronically recorded by two independent study nurses.

#### Study risk of bias assessment

The Cochrane Collaboration Cochrane Risk of Bias Tool (RoB) (45) was used to assess the internal validity of the included articles. Two independent reviewers (SR, JT) assessed the methodological quality of the eligible studies with “The Cochrane Collaboration's tool for assessing risk of bias”. The criteria list comprised six items and each item were scored with + for yes, with—for no, and with? if the information was not provided or was unclear. A study was determined as having a low risk of bias if all criteria are fulfilled with yes. A study has a moderate risk of bias when one or more items are scored with unclear, while a study has a high risk of bias if one or more key domains have been rated with no. The level of agreement between the independent reviewers who rated the primary studies was 98%.

## Results

### Study selection

**Figure 1:**
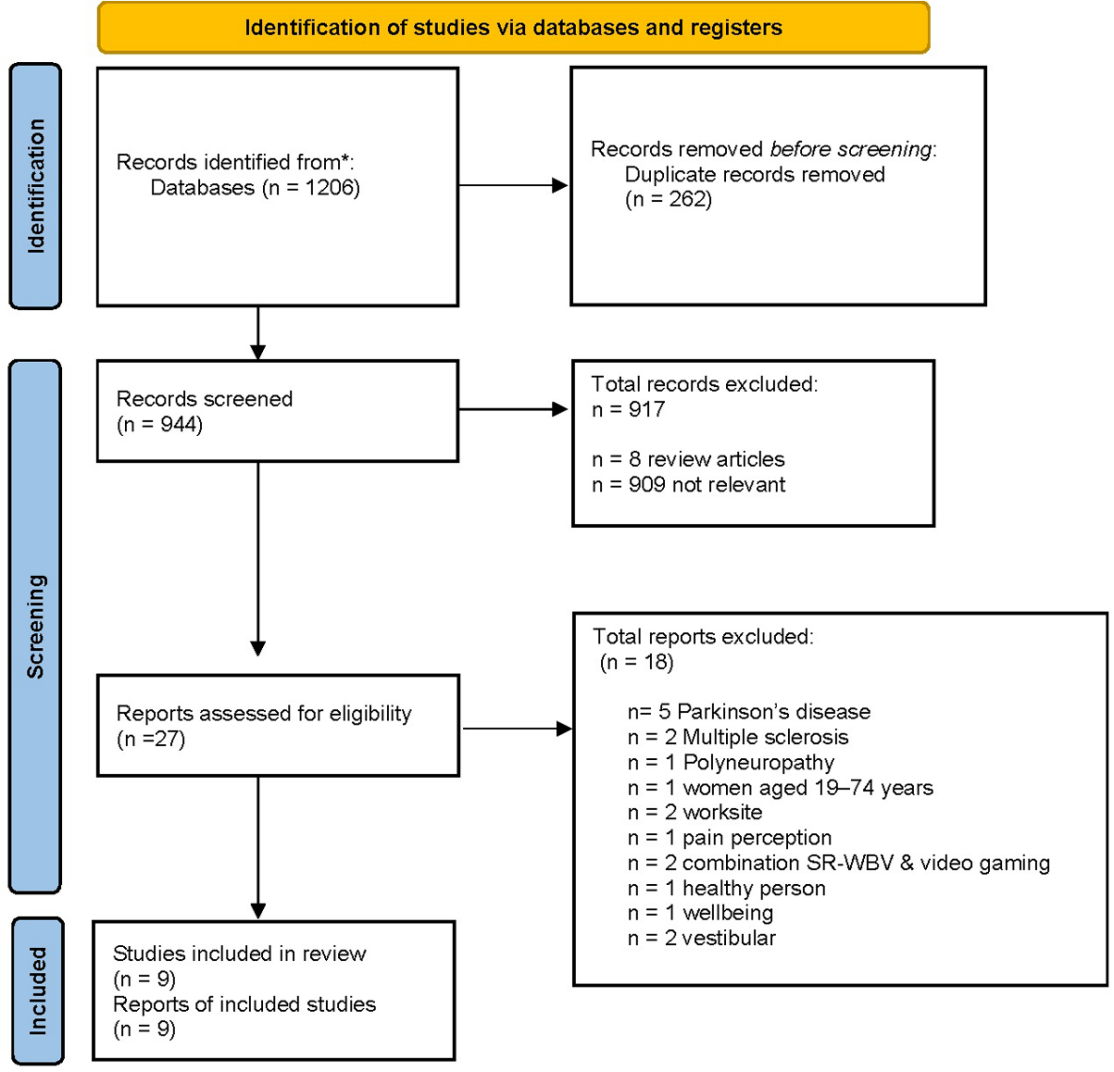
Flow chart of the search.

There were 1,206 matches of studies. Of these, 262 duplicates were removed. A total of 944 titles and abstracts were screened, and 917 articles were removed due to systematic reviews articles (*n* = 8), application of sinusoidal vibration (*n* = 898), application of stochastic vibration *via* the sole of the foot or knee (*n* = 7), effects of SR-WBV on postural control or pain (*n* = 2), pelvic floor muscle (*n* = 2). The remaining 27 full texts were read, of which 9 articles were included in this systematic review (Figure 1). Three articles originated from Germany (4, 46, 47), and six from Switzerland^1^ (16, 19, 30, 36, 48, 49). Six trials were designed as pilot study (16, 19, 30, 36, 48, 49) and three as randomized controlled trials (4, 46, 47).

### Balance survey method

A total of six articles examined static balance (16, 19, 30, 47–49), six studies examined dynamic balance (4, 16, 19, 30, 47, 49) while five studies observed functional balance (19, 46–49).

### Result overview of the studies

Overall, four of nine studies showed statistically significant balance improvements within the SR-WBV group in the before-after comparison (4, 19, 46, 48). Table 1 summarizes the findings of the individual articles.

**Table 1:** Overview of the study characteristics of the included studies.

Study	Population	Intervention protocol	Target parameters		Results	
	Group: SR-WBV; intervention group (IG); control group (CG), (n), sex (men (m)/women (w)), mean years (±SD)		Biomechanical measurement method	Functional parameter/outcome	Effect within group (effect size: ES)	Effect between groups
Dittrich et al. (47)	Prospective controlled study SR-WBV: *n* = 41 (m: 13/ w: 28) CG: *n* = 52 (m: 13 / w: 39) SR-WBV: m: 69.1(±4.0) w: 66.4(±5.0) CG: m: 68.1 (±6.8)/ w: 70.0 (±7.5)	WBV: Exercises on ZeptorMed (4 exercises per training session from a pool of 15; individual, not listed which exercises). IG: activity in everyday life was not changed	Static & dynamic balance with Biodex Stability System	Dynamic, functional balance with motoric assessment according Runge [Chair Rising, Timed-up-and-Go (TUG), Tandem walk, etc.]	IG (women) Chair Rising significantly decreased by 0.9 s (*p* = 0.003; ES = 0.4) TUG significantly reduced by 0.4 s (*p* = 0.000; ES = 0.4)	
Haas et al. (46)	RCT cross-over Group A: *n* = 34 Group B: *n* = 34 m: 53/w: 15 65.0 years (±7.8)	SR-WBV: Free two leg stand with shoes, with slightly bent knees CG: rest for the same duration		Static and functional balance with UPDRS motor scores und Sit-to-Stand (STS)	Significant reduction in UPDRS motor score (*p* < 0.01) after SR-WBV Group A: - 16.8% Group B: - 14.7%	
Kessler et al. (48)	RCT pilot study SR-WBV: *n* = 13 (m: 5/w: 8) CG: *n* = 11 (m: 3/w: 8) SR-WBV: 90.7 years (±7.5) KG: 83.8 years (±9.3)	SR-WBV: Parallel stance (increase possible without holding), tandem stance, slow dynamic squats CG: Sham therapy without increasing 1 Hz		Static and functional balance with chair rise Test during Short Physical Performance Battery test (SPPB)	Chair rising significantly reduced (*p* = 0.001; ES r = 0.89)	Significantly higher SPPB score significantly for SR-WBV compared to CG (*p* = 0.035; ES r = 0.43)
Rogan et al. (16)	RCT crossover pilot study Group A: *n* = 10 Group B: *n* = 10 Group A: 76.8 years (±7.7) Group B: 80.7 years (±5.7)	Parallel stance with shoes with slightly bent knees	Static balance with Kistler force plate	Dynamic balance with functional reach test FRT; Expanded Timed get Up and Go Test (ETGUG); (Single-task / Dual-task)		
Rogan et al. (49)	RCT crossover pilot study SR-WBV: *n* = 9 (m/w: n.i.) SR-GKV: 88.5 Jahre (±6)	Parallel stance with shoes with slightly bent knees		Static, dynamic and functional balance with semitandem/tandem stand & Chair Rise Test (during SPPB Test), ETGUG	Large ES in SPPB score after SR-WBV (*p* = 0.039)	Large ES for ETGUG (*p* = 0.043)
Rogan et al. (30)	RCT crossover pilot study SR-GKV: *n* = 9 (m:4/w:4) SR-GKV: 88.5 Jahre (±5.9)	Parallel stance with shoes with slightly bent knees		Static, dynamic balance by ETGUG, chair rising	Large ES for SPPB (*p* = 0.121); ETGUG (*p* = 0.011);	
Rogan et al. (19)	RCT crossover pilot study SR-WBV: *n* = 10 (m: 5/w: 5) CG: *n* = 10 (m:5/w:5) SR-WBV: 80.2 years (±6.8) CG: 77.4 years (±7.1)	SR-WBV: Parallel stance with shoes with slightly bent knees KG: Sham intervention with 1 Hz; same position as SR-WBV		Static, dynamic and functional balance with semitandem/tandem stand & Chair Rise Test (during SPPB Test), ETGUG		
Rogan et al. (36)	RCT crossover pilot studie SR-WBV: *n* = 10 (m/w: n. i.) CG: *n* = 10 (m/w: n.i.) SR-WBV: 76.8 years (±7.7) KG: 80.7 years (±5.7)	SR-WBV: Parallel stance with shoes with slightly bent knees KG: Sham intervention with 1 Hz; same position as SR-WBV	Functional balance by chair rising on a Kistler force plate		Significantly faster rising (*p* = 0.09)	
Turbanski et al. (4)	Case-control-study SR-WBV: *n* = 26 (m/w: n.i.) CG: *n* = 26 (m/w: n. i.) *n* = 52 (m: 38/w: 14) *n* = 69.1 years (± 8.9)	SR-WBV: Upright standing on ZeptorMed CG: 15 min walk	Dynamic balance by means of TS and narrow PS on moving platform		Tandem stance was significantly longer after SR-WBV over 20 s (*p* = 0.04)	

### Training loads

In four of the nine studies, participants received a single training session with SR-WBV (4, 19, 30, 46). The remaining five studies determined the effect of SR-WBV after multiple interventions. The range was 12–36 training sessions (16, 36, 47–49).

In three studies (4, 46, 48), the frequency was increased and the starting position on the vibration device was progressively adjusted to the participants. All three studies use 5 sets and 60 s of vibration. One study did not specify a frequency (47). In three trials, continuous vibration was performed at a frequency of 5 Hz and five series with a duration of 60 s and rest of 60 s (19, 36). Four studies applied a frequency of 6 Hz, with 5–6 series of 60 s duration and 60 s rests (4, 16, 30, 46, 49).

The control group (CG) received no active intervention in four out of nine studies (19, 46, 47, 50). In one study, the CG completed a different intervention (4). Sham intervention was performed in four other studies. Table 2 gives an overview of the training load.

**Table 2:** SR-WBV training load as used in the different studies under investigation.

Study	Duration / (sessions per week)	Frequency	Sets, duration, rest
Dittrich et al. (47)	12 weeks / (3×)	n. i.	3 × 45–60 s, 3 s
Haas et al. (46)	1 day / (1×)	6 Hz (±1 Hz)	5 × 6 s, 6 s
Kessler et al. (48)	4 weeks / (3×)	3–6 Hz	5 × 6 s, 6 s
Rogan et al. (16)	2 × 4 weeks / (3×)	5 Hz	5 × 6 s, 6 s
Rogan et al. (49)	2 × 4 weeks / (3×)	6 Hz	5 × 6 s, 6 s
Rogan et al. (30)	1 day / (1×)	6 Hz	6 × 6 s, 6 s
Rogan et al. (19)	1 day / (1×)	5 Hz	5 × 6 s, 6 s
Rogan et al. (36)	4 weeks / (3×)	5 Hz	5 × 6 s, 6 s
Turbanski et al. (4)	1 day / (1×)	6 Hz (±1 Hz)	5 × 6 s, n. i.

n. i., no indication; h, hertz; s, seconds.

The evaluation of the methodological quality was classified as followed (Table 3): one study did not use the method of allocation concealment (47), seven studies (4, 16, 19, 30, 46, 47, 49) did not report the blinded status of the investigator or participant, and four studies (4, 19, 46, 49) showed incomplete outcome data. They presented only change percentage data. They did not give any information about baseline and intervention data and no effect size calculation was used. Table 3 provides an overview of the risk of bias of the included studies.

**Table 3:** Risk of bias of the included studies.

	Random sequence generation (selection bias)	Allocation concealment (selection bias)	Blinding of participants (performance bias)	Blinding of personnel (detection bias)	Incomplete outcome data (attrition bias)	Selective reporting (reporting bias)	Other bias
Dittrich et al. (47)	?	–	–	+	?	?	–
Haas et al. (46)	?	?	–	+	–	+	–
Kessler et al. (48)	+	+	–	–	+	+	+
Rogan et al. (30)	+	+	+	?	?	–	+
Rogan et al. (16)	+	+	+	–	+	+	+
Rogan et al. (49)	+	+	–	–	–	–	–
Rogan et al. (19)	+	+	–	–	–	–	+
Rogan et al. (36)	+	+	–	–	+	+	–
Turbanski et al. (4)	?	?	?	?	–	+	–

Bias rating: (+) = there is a small risk of bias; (–) = there is a risk of bias; (?) = unclear bias because not enough information available.

## Discussion

During aging, sensory symptoms such as absent reflexes are clinically relevant. They are not only debilitating but also responsible for changes. This systematic literature review addressed the research question “could SR-WBV positively influence postural control in individuals with balance disability?” using published results.

In summary, the effect of SR-WBV on postural control presents a mixed picture. The statistically significant results from four studies were contrasted with five statistically unsound results. However, the effect size was strengthened by Rogan et al.'s (49) indication of clinical relevance. They were able to demonstrate clinical relevance (2.9 s) for the Expanded Timed Get-up-and-Go (ETGUG) test (51) in frail individuals after SR-WBV training. The SR-WBV group was a median of 3 s faster after the intervention period compared with the baseline measurement (*P* = 0.043; ES: 0.91). This study result has immediate consequences in terms of treatment recommendation for frail individuals with a postural control deficit of dynamic balance (52). It is known that a normal sensory system is necessary for successful postural control and movement. The central nervous system must accurately assess the position of the body in space and the limbs in relation to each other (proprioception). Postural reflexes must be released efficiently when external perturbations are detected. Maintaining balance must be automatic so that it is not impaired by other tasks. During aging, impairments of the sensorimotor system lead to a loss of postural control and to falls. The process of postural control depends on many sensory signals and neurological pathways and maintaining the quality of these systems at their optimal level is fundamental. SR-WBV could play an important role toward addressing postural control, by involving an interaction of different types of neurophysiological sensors and the adaptation of afferent and efferent signals, the SR-WBV likely serves as an exercise for the sensorimotor system. Tan et al. (53) demonstrated in their systematic review a significant positive benefit on postural control (SMD = 0.61, 95% CI: 0.12 to 1.09, *P* = 0.01) and muscle activity in tibialis anterior (SMD = 0.46, 95% CI: 0.04 to 0.88, *P* = 0.03) and gastrocnemius (SMD = 0.68, 95% CI: 0.14 to 1.23, *P* = 0.01) using sinusoidal whole-body vibration in individuals with a sensorimotor deficit after ankle injury. They concluded that whole body vibration has the potential to improve sensorimotor deficits involving balance, strength, joint position sense, and muscle activity in people with chronic ankle instability. However, Lesinski et al. (54) formulated in their systematic review and meta-analysis article a balance training regime for healthy elderly by a training period of 11–12 weeks, a training frequency of three sessions per week, a total number of 36–40 training sessions, a duration of 31–45 min of a single training session, and a total duration of 91–120 min of balance training per week. Comparing these findings with findings from young healthy adults, it seems plausible that almost the same balance protocols are effective in healthy young and older adults and there seems to be no age effect (54). In this current article, no of the included articles reported this amount of training regimes. Fisher et al. were able to illustrate in their meta-analysis, that long-term WBV (between 4 weeks and 32 weeks) could significantly improve functional balance (Timed-up-Go test: SMD = −0.18; 95% CI: −0.32, −0.04; 10 min walking test SMD = −0.28; 95% CI: −0.56, −0.01). However, no significant changes were found in elderly individuals (tinetti gait scores: SMD = 0.04; 95% CI: −0.23, 0.31, 6 min walking test: SMD = 0.37; 95% CI: −0.03, 0.78).

It is known that muscle strength is a potentially important factor contributing to postural control (61). Large effects of strength training could be determined for static and dynamic balance in elderly individuals, but only a small effect was found for dynamic balance in young adults (62). Son et al. (63) were able to demonstrate that strength training increase muscle strength in ankle musculature and improve one-leg-standing balance compared to control situation. It can be concluded that the intensity of strength training is fundamental not only for increasing muscle strength but also for improving postural balance in elderly participants.

Furthermore, Kingwell described that exercise has the potential to improve cognitive function (64). Explanatory models address the fact that WBV stimulate mechanosensory receptors (e.g., tactile corpuscles). These signals are transmitted to the primary somatosensory cortex. These areas have connection with region in the prefrontal cortex that strongly involved in cognitive processing (37, 65). An indirect pathway involves the limbic system (e.g., amygdala and hippocampus, important areas of learning and memory), which can mediate the effects of sensory correlations on the prefrontal cortex (66). The amygdala has projections to non-thalamic nuclei (e.g., the cholinergic nuclei of the basal forebrain) with diffuse connections to several brain regions (65). It can be speculated that mechanosensory receptor stimulation can increase cognitive function. Furthermore, it has been assumed that improvement in cognitive function depends on increased production of neurotrophins [e.g., brain-derived neurotrophic factor (BDNF)] (67). BDNF is recognized as the most significant neurotrophic growth factor related to neuronal plasticity and has a key role in the differentiation and survival of neurons (68). Studies could demonstrate a close correlation between increased BDNF levels and WBV (69, 70). However, so far it is unclear how mechanical vibrations may influence the expression of BDNF (71).

The loss of balance ability is an important risk factor for falls in elderly individuals. Reactive balance is a crucial part of avoiding and adapting to complex environments that threaten postural stability (72). In German-speaking countries, the balance ability is considered to be a coordinative ability (73). We describe this ability as the aggregate understood to maintain and regain balance, taking into account the necessary personal conditions.

Various types of exercises (e.g., airex pad, tilting board, swinging platforms) are used in treatment settings and summarized with the synonym balance training (74–77). The goal is to optimize balance. It is assumed that balance is a skill, and that balance training improves several balance tasks at the same time (29). However, recent studies indicate that only those balance tasks that are trained can also improve (77, 78). Giboin et al. (32) showed that the group which trained in a single-leg stand on the tilting board and the group that trained in a single-leg stand on the swinging board (Posturomed) improved statistically significantly only in the area in which they trained.

Recently, there have been attempts to move away from the term ability towards the definition of skill (73). Taube (78) explained that balance training does not change the behavior of the spinal reflex *per se*. It seems rather to improve the ability of finding the right reflex settings for specific conditions of postural control. Thus, balance training improves task-specific reflex modulation. Low et al. (55) postulated, that specific balance exercise could be the only one likely to improve postural balance. Slackline training improves postural balance in young and elderly individuals in a one-leg stance (56, 57). However, the impact of slackline training is limited or negligible for standard static and dynamic bipedal stances (58–60). Paillard (79) explains that specific balance training optimizes postural skills, but it is not known whether these skills improve motor skills in all types of physical activity. He further refers to the fact that additional studies are required to address this question accurately. Grabiner et al. (80) indicates that task-specific perturbation training is superior to traditional balance exercise training in improving reactive balance capacity and thus preventing falls. Kim et al. (72) performed a network meta-analysis to specify which exercise method is most effective to improve reactive balance in elderly individuals. They analyzed data of 39 RCTs including 1,388 elderly individuals receiving balance training with reactive components (perturbations training) demonstrated the most amount of improvement in reactive training, followed by power training and gait training. SR-WBV is power training. SR-WBV has the potential to improve race of force development after four weeks SR-WBV training in elderly individuals (48). In relation to gait, SR-WBV can be used as skilling up in elderly not able to perform standard gait training. It is known that SR-WBV could significantly improve gait in older adults (19, 47, 49). In the case of a reactive balance, the better the gait, the sooner gait training can be started.

## Limitation

The observed heterogeneity in the individual studies' study quality and findings impede a clear-cut answer to the research question. Furthermore, the study design of the pilot study does not allow a clear conclusion on efficacy because the primary aim of the pilot study is not to assess an exact intervention effect size, but rather to determine the sample sizes and evaluate feasibility of the study protocol (81–83).

## Conclusion

We found a heterogeneous situation on effects for balance according to SR-WBV. One study showed clinical relevance for ETGUG. Two studies examined the skill in reactive balance. Since balance is a skill and SR-WBV trains reactive balance, future studies should focus on the parameter reactive balance.

## Data Availability

The original contributions presented in the study are included in the article/Supplementary Material, further inquiries can be directed to the corresponding author/s.

## Footnotes

^1^These articles are from the authors of this systematic review.

## References

[1] Muslimović D Post B Speelman JD Schmand B de Haan RJ. Determinants of disability and quality of life in mild to moderate Parkinson disease. Neurology. (2008) 70(23):2241. 10.1212/01.wnl.0000313835.33830.80 18519873

[2] Faes Y Rolli Salathé C Herlig ML Elfering A. Beyond physiology: acute effects of side-alternating whole-body vibration on well-being, flexibility, balance, and cognition using a light and portable platform A randomized controlled trial. Front Sports Act Living. (2023) 5. 10.3389/fspor.2023.1090119 36793620 PMC9922907

[3] Priplata AA Niemi JB Harry JD Lipsitz LA Collins JJ. Vibrating insoles and balance control in elderly people. Lancet. (2003) 362(9390):1123–4. 10.1016/S0140-6736(03)14470-4 14550702

[4] Turbanski S Haas CT Schmidtbleicher D Friedrich A Duisberg P. Effects of random whole-body vibration on postural control in Parkinson’s disease. Res Sports Med. (2005) 13(3):243–56. 10.1080/15438620500222588 16392539

[5] Schuhfried O Mittermaier C Jovanovic T Pieber K Paternostro-Sluga T. Effects of whole-body vibration in patients with multiple sclerosis: a pilot study. Clin Rehabil. (2005) 19(8):834–42. 10.1191/0269215505cr919oa 16323382

[6] Herren K Schmid S Rogan S Radlinger L. Effects of stochastic resonance whole-body vibration in individuals with unilateral brain lesion: a single-blind randomized controlled trial: whole-body vibration and neuromuscular function. Rehabil Res Pract. (2018) 2018. 10.1155/2018/9319258 30155308 PMC6093017

[7] Priplata AA Patritti BL Niemi JB Hughes R Gravelle DC Lipsitz LA Noise-enhanced balance control in patients with diabetes and patients with stroke. Ann Neurol. (2006) 59(1):4–12. 10.1002/ana.20670 16287079

[8] Collins JJ Imhoff TT Grigg P. Noise-enhanced information transmission in rat SA1 cutaneous mechanoreceptors via aperiodic stochastic resonance. J Neurophysiol. (1996) 76(1):642–5. 10.1152/jn.1996.76.1.642 8836253

[9] Cordo P Inglis JT Verschueren S Collins JJ Merfeld DM Rosenblum S Noise in human muscle spindles. Nature. (1996) 383(6603):769–70. 10.1038/383769a0 8892999

[10] Douglass JK Wilkens L Pantazelou E Moss F. Noise enhancement of information transfer in crayfish mechanoreceptors by stochastic resonance. Nature. (1993) 365(6444):337–40. 10.1038/365337a0 8377824

[11] Levin JE. Miller JP. Broadband neural encoding in the cricket cereal sensory system enhanced by stochastic resonance. Nature. (1996) 380(6570):165–8. 10.1038/380165a0 8600392

[12] Collins JJ Imhoff TT Grigg P. Noise-enhanced tactile sensation. Nature. (1996) 383(6603):770. 10.1038/383770a0 8893000

[13] Collins JJ Chow CC Imhoff TT. Stochastic resonance without tuning. Nature. (1995) 376:236–8. 10.1038/376236a0 7617033

[14] Wiesenfeld K Moss F. Stochastic resonance and the benefits of noise: from ice ages to crayfish and SQUIDs. Nature. (1995) 373(6509):33–6. 10.1038/373033a0 7800036

[15] Herren K Hängärtner CH Oberli A Radlinger L. Kardiovaskuläre und metabolische Beanspruchung während stochastischer Resonanztherapie bei Schlaganfallpatienten. Physioscience. (2009) 5(01):13–7. 10.1055/s-0028-1109140

[16] Rogan S Radlinger L Hilfiker R Schmidtbleicher D De Bie RA De Bruin ED. Feasibility and effects of applying stochastic resonance whole-body vibration on untrained elderly: a randomized crossover pilot study. BMC Geriatr. (2015) 15(1):25. 10.1186/s12877-015-0021-4 25886789 PMC4371632

[17] Skelton DA Dinan SM. Exercise for falls management: rationale for an exercise programme aimed at reducing postural instability. Physiother Theory Pract. (1999) 15(2):105–20. 10.1080/095939899307801

[18] Rogan S. Innovative training programs for frail elderly in the skilling up stage. (2016).

[19] Rogan S Radlinger L Schmid S Herren K Hilfiker R de Bruin ED. Skilling up for training: a feasibility study investigating acute effects of stochastic resonance whole-body vibration on postural control of older adults. Ageing Res. (2012) 3(1):e5-e. 10.4081/ar.2012.e5

[20] Ritzmann R Kramer A Bernhardt S Gollhofer A. Whole body vibration training-improving balance control and muscle endurance. PLoS One. (2014) 9(2):e89905. 10.1371/journal.pone.0089905 24587114 PMC3935964

[21] Eichelberger P Fankhauser R Geering R Radlinger L Rogan S. Trunk muscle activity and acceleration of the spine during partial-body vibration in a sitting position—a single case study. Physiotherapy. (2015) 101:e347. 10.1016/j.physio.2015.03.556

[22] Johanning E. Whole-body vibration-related health disorders in occupational medicine–an international comparison. Ergonomics. (2015) 58(7):1239–52. 10.1080/00140139.2015.1005170 25655650

[23] Scherrer A. Risikofaktor vibrationen: So schützen sie die gesundheit ihrer mitarbeitenden. SUVA: SUVA- Gesundheitschutz (2012).

[24] Rogan S Taeymans J Radlinger L Naepflin S Ruppen S Bruelhart Y Effects of whole-body vibration on postural control in elderly: an update of a systematic review and meta-analysis. Arch Gerontol Geriatr. (2017) 73:95–112. 10.1016/j.archger.2017.07.022 28800481

[25] Rogan S de Bruin ED Radlinger L Joehr C Wyss C Stuck N-J Effects of whole-body vibration on proxies of muscle strength in old adults: a systematic review and meta-analysis on the role of physical capacity level. Eur Rev Aging Phys Act. (2015) 12(1):1–26. 10.1186/s11556-015-0149-4 26865876 PMC4748331

[26] Rogan S Hilfiker R Herren K Radlinger L de Bruin ED. Effects of whole-body vibration on postural control in elderly: a systematic review and meta-analysis. BMC Geriatr. (2011) 11(1):1–18. 10.1186/1471-2318-11-72 22054046 PMC3229447

[27] Rogan S Hilfiker R Schenk A Vogler A Taeymans J. Effects of whole-body vibration with stochastic resonance on balance in persons with balance disability and falls history–a systematic review. Res Sports Med. (2014) 22(3):294–313. 10.1080/15438627.2014.919504 24950116

[28] Gammaitoni L Hänggi P Jung P Marchesoni F. Stochastic resonance. Rev Mod Phys. (1998) 70(1):223. 10.1103/RevModPhys.70.223

[29] Haas CT Turbanski S Markitz S Kaiser I Schmidtbleicher D. Stochastische Resonanz in der Therapie von Bewegungsstörungen. B&G Bewegungstherapie und Gesundheitssport. (2006d) 22:58–61. 10.1055/s-2006-933388

[30] Rogan S Schmidtbleicher D Radlinger L. Immediate effects after stochastic resonance whole-body vibration on physical performance on frail elderly for skilling-up training: a blind cross-over randomised pilot study. Aging Clin Exp Res. (2014) 26(5):519–27. 10.1007/s40520-014-0212-4 24700493

[31] Krause A Freyler K Gollhofer A Stocker T Brüderlin U Colin R Neuromuscular and kinematic adaptation in response to reactive balance training–a randomized controlled study regarding fall prevention. Front Physiol. (2018) 9:1075. 10.3389/fphys.2018.01075 30131722 PMC6090079

[32] Palmer JA Payne AM Ting LH Borich MR. Cortical engagement metrics during reactive balance are associated with distinct aspects of balance behavior in older adults. Front Aging Neurosci. (2021) 13:684743. 10.3389/fnagi.2021.684743 34335230 PMC8317134

[33] Rogan S Radlinger L. Sturzprävention: mehr Sicherheit. Med Move. (2015):32–4.

[34] De Bruin ED Baur H Brülhart Y Luijckx E Hinrichs T Rogan S. Combining stochastic resonance vibration with exergaming for motor-cognitive training in long-term care; A sham-control randomized controlled pilot trial. Front Med (Lausanne). (2020) 7:507155. 10.3389/fmed.2020.507155 33330519 PMC7734185

[35] Rogan S Radlinger L Baur H Schmidtbleicher D de Bie RA de Bruin ED. Sensory-motor training targeting motor dysfunction and muscle weakness in long-term care elderly combined with motivational strategies: a single blind randomized controlled study. Eur Rev Aging Phys Act. (2016) 13(1):1–12. 10.1186/s11556-016-0164-0 27239241 PMC4884400

[36] Rogan S Hilfiker R Schmid S Radlinger L. Stochastic resonance whole-body vibration training for chair rising performance on untrained elderly: a pilot study. Arch Gerontol Geriatr. (2012) 55(2):468–73. 10.1016/j.archger.2012.02.011 22425243

[37] Regterschot GRH Van Heuvelen MJG Zeinstra EB Fuermaier ABM Tucha L Koerts J Whole body vibration improves cognition in healthy young adults. PLoS One. (2014) 9(6):e100506. 10.1371/journal.pone.0100506 24949870 PMC4065066

[38] Timmer M Van der Zee EA Riedel G. Whole body vibration and behavior: Investigation of the role of various neurotransmitter systems. Federation of European Neuroscience Societies Abstract. (2006) 3(089.31)

[39] Van der Zee EA Riedel G Rutgers EH De Vries C Postema F Venema BJ. Enhanced neuronal activity in selective brain regions of mice induced by whole body stimulation. Federation of European Neuroscience Societies Abstract. (2010) 5(024.49):R2.

[40] Chan RCK Shum D Toulopoulou T Chen EYH. Assessment of executive functions: review of instruments and identification of critical issues. Arch Clin Neuropsychol. (2008) 23(2):201–16. 10.1016/j.acn.2007.08.010 18096360

[41] Chen TY Peronto CL Edwards JD. Cognitive function as a prospective predictor of falls. J Gerontol B Psychol Sci Soc Sci. (2012) 67(6):720–8. 10.1093/geronb/gbs052 22865822 PMC3636670

[42] Springer S Giladi N Peretz C Yogev G Simon ES Hausdorff JM. Dual-tasking effects on gait variability: the role of aging, falls, and executive function. Mov Disord. (2006) 21(7):950–7. 10.1002/mds.20848 16541455

[43] Tinetti ME. Preventing falls in elderly persons. N Engl J Med. (2003) 348(1):42–9. 10.1056/NEJMcp020719 12510042

[44] Doumas M Rapp MA Krampe RT. Working memory and postural control: adult age differences in potential for improvement, task priority, and dual tasking. J Gerontol B. (2009) 64(2):193–201. 10.1093/geronb/gbp009 PMC2655174 19255088

[45] Higgins JPT Altman DG Gøtzsche PC Jüni P Moher D Oxman AD The cochrane collaboration’s tool for assessing risk of bias in randomised trials. Br Med J. (2011) 343. 10.1136/bmj.d5928 PMC3196245 22008217

[46] Haas CT Turbanski S Kessler K Schmidtbleicher D. The effects of random whole-body-vibration on motor symptoms in Parkinson’s disease. Neurorehabilitation. (2006b) 21:29–36. 10.3233/NRE-2006-21105 16720935

[47] Dittrich M Eichner G Schmidtbleicher D Beyer WF. Eine Untersuchung zur Wirkung der Stochastischen Resonanztherapie (SRT-Zeptoring) auf die Knochendichte, Rumpfkraft und Koordination bei Senioren. OUP. (2012) 1(2):60–5. 10.328/oup.2012.0060-0065

[48] Kessler J Radlinger L Baur H Rogan S. Effect of stochastic resonance whole body vibration on functional performance in the frail elderly: a pilot study. Arch Gerontol Geriatr. (2014) 59(2):305–11. 10.1016/j.archger.2014.06.005 25042993

[49] Rogan S Radlinger L Schmidtbleicher D de Bie RA de Bruin ED. Preliminary inconclusive results of a randomised double blinded cross-over pilot trial in long-term-care dwelling elderly assessing the feasibility of stochastic resonance whole-body vibration. Eur Rev Aging Phys Act. (2015) 12(1):5. 10.1186/s11556-015-0150-y 26865869 PMC4745146

[50] Haas CT Buhlmann A Turbanski S Schmidtbleicher D. Proprioceptive and sensorimotor performance in Parkinson’s disease. Res Sports Med. (2006a) 14. 10.1080/15438620600985902 17214404

[51] Botolfsen P Helbostad JL Moe-nilssen R Wall JC. Reliability and concurrent validity of the expanded timed up-and-go test in older people with impaired mobility. Physiother Res Int. (2008) 13(2):94–106. 10.1002/pri.394 18288773

[52] White O Babič J Trenado C Johannsen L Goswami N. The promise of stochastic resonance in falls prevention. Front Physiol. (2019) 9. 10.3389/fphys.2018.01865 30745883 PMC6360177

[53] Tan J Wu X Clark CCT Barton V Chen S Liu S The effect of whole body vibration on sensorimotor deficits in people with chronic ankle instability: a systematic review and meta-analysis. Clin Rehabil. (2022) 36(8):1016–31. 10.1177/02692155221095 35535548

[54] Lesinski M Hortobágyi T Muehlbauer T Gollhofer A Granacher U. Effects of balance training on balance performance in healthy older adults: a systematic review and meta-analysis. Sports Med. (2015) 45(12):1721–38. 10.1007/s40279-015-0375-y 26325622 PMC4656699

[55] Horlings CGC Van Engelen BG Allum JHJ Bloem BR. A weak balance: the contribution of muscle weakness to postural instability and falls. Nat Clin Pract Neurol. (2008) 4(9):504–15. 10.1038/ncpneuro0886 18711425

[56] Behm DG Muehlbauer T Kibele A Granacher U. Effects of strength training using unstable surfaces on strength, power and balance performance across the lifespan: a systematic review and meta-analysis. Sports Med. (2015) 45:1645–69. 10.1007/s40279-015-0384-x 26359066 PMC4656700

[57] Son SM Kang KW Lee NK Nam SH Kwon JW Kim K. Influence of isokinetic strength training of unilateral ankle on ipsilateral one-legged standing balance of adults. J Phys Ther Sci. (2013) 25(10):1313–5. 10.1589/jpts.25.1313 24259783 PMC3820187

[58] Kingwell K. An exercise-linked mediator of memory protection. Nat Rev Drug Discovery. (2019) 18(2):97–8. 10.1038/d41573-019-00006-x 30710134

[59] Braak H Braak E Yilmazer D Bohl J. Topical review: functional anatomy of human hippocampal formation and related structures. J Child Neurol. (1996) 11(4):265–75. 10.1177/088307389601100402 8807415

[60] Durgut E Orengul AC Algun ZC. Comparison of the effects of treadmill and vibration training in children with attention deficit hyperactivity disorder: a randomized controlled trial. NeuroRehabilitation. (2020) 47(2):121–31. 10.3233/NRE-203040 32741784

[61] Bonanni R Cariati I Tarantino U D’Arcangelo G Tancredi V. Physical exercise and health: a focus on its protective role in neurodegenerative diseases. J Funct Morphol Kinesiol. (2022) 7(2):38. 10.3390/jfmk7020038 35645300 PMC9149968

[62] von Bohlen und Halbach O von Bohlen und Halbach V. BDNF Effects on dendritic spine morphology and hippocampal function. Cell Tissue Res. (2018) 373:729–41. 10.1007/s00441-017-2782-x 29450725

[63] Ribeiro VGC Lacerda ACR Santos JM Coelho-Oliveira AC Fonseca SF Prates ACN Efficacy of whole-body vibration training on brain-derived neurotrophic factor, clinical and functional outcomes, and quality of life in women with fibromyalgia syndrome: a randomized controlled trial. J Healthc Eng. (2021) 2021. 10.1155/2021/7593802 34900203 PMC8654532

[64] Simão AP Mendonça VA Avelar NCP da Fonseca SF Santos JM De Oliveira ACC Whole body vibration training on muscle strength and brain-derived neurotrophic factor levels in elderly woman with knee osteoarthritis: a randomized clinical trial study. Front Physiol. (2019) 10:756. 10.3389/fphys.2019.00756 31293437 PMC6603338

[65] Bonanni R Cariati I Romagnoli C D’Arcangelo G Annino G Tancredi V. Whole body vibration: a valid alternative strategy to exercise? J Funct Morphol Kinesiol. (2022) 7(4):99. 10.3390/jfmk7040099 PMC9680512 36412761

[66] Kim Y Vakula MN Bolton DAE Dakin CJ Thompson BJ Slocum TA Which exercise interventions can most effectively improve reactive balance in older adults? A systematic review and network meta-analysis. Front Aging Neurosci. (2022) 13:992. 10.3389/fnagi.2021.764826 PMC8804322 35115917

[67] Kramer A Giboin L-S. Gleichgewichtstraining: transfer auf untrainierte Aufgaben? Sportphysio. (2019) 7(01):16–21. 10.1055/a-0818-3129

[68] Rogan S Baur H Sargent A Schori M Taeymans J. Machbarkeit eines Gleichgewichtstrainings auf Matten bei gesunden, moderat sportlichen Frauen im Alter. Z Gerontol Geriat. (2015) 48(2):135–41. 10.1007/s00391-014-0630-0 24659024

[69] Heitkamp HC Horstmann T Mayer F Weller J Dickhuth HH. Balance training in men and women: effect on knee extensors and flexors. Isokinet Exerc Sci. (2001) 9(1):41–4. 10.3233/IES-2001-0062

[70] Giboin L-S Gruber M Kramer A. Task-specificity of balance training. Hum Mov Sci. (2015) 44:22–31. 10.1016/j.humov.2015.08.012 26298214

[71] Kümmel J Kramer A Giboin L-S Gruber M. Specificity of balance training in healthy individuals: a systematic review and meta-analysis. Sports Med. (2016) 46(9):1261–71. 10.1007/s40279-016-0515-z 26993132

[72] Taube W. Neurophysiological adaptations in response to balance training. German J Sports Med. (2012) 63(9). 10.5960/dzsm.2012.030

[73] Low DC Walsh GS Arkesteijn M. Effectiveness of exercise interventions to improve postural control in older adults: a systematic review and meta-analyses of centre of pressure measurements. Sports Med. (2017) 47:101–12. 10.1007/s40279-016-0559-0 27245061 PMC5215248

[74] Thomas M Kalicinski M. The effects of slackline balance training on postural control in older adults. J Aging Phys Act. (2016) 24(3):393–8. 10.1123/japa.2015-0099 26583953

[75] Pfusterschmied J Stöggl T Buchecker M Lindinger S Wagner H Müller E. Effects of 4-week slackline training on lower limb joint motion and muscle activation. J Sci Med Sport. (2013) 16(6):562–6. 10.1016/j.jsams.2012.12.006 23333134

[76] Granacher U Iten N Roth R Gollhofer A. Slackline training for balance and strength promotion. Int J Sports Med. (2010) 31(10):717–23. 10.1055/s-0030-1261936 20677124

[77] Donath L Roth R Zahner L Faude O. Slackline training and neuromuscular performance in seniors: a randomized controlled trial. Scand J Med Sci Sports. (2016) 26(3):275–83. 10.1111/sms.12423 25756231

[78] Donath L Roth R Zahner L Faude O. Slackline training (balancing over narrow nylon ribbons) and balance performance: a meta-analytical review. Sports Med. (2017) 47(6):1075–86. 10.1007/s40279-016-0631-9 27704483

[79] Paillard T. Plasticity of the postural function to sport and/or motor experience. Neurosci Biobehav Rev. (2017) 72:129–52. 10.1016/j.neubiorev.2016.11.015 27894829

[80] Grabiner MD Crenshaw JR Hurt CP Rosenblatt NJ Troy KL. Exercise-based fall prevention: can you be a bit more specific? Exerc Sport Sci Rev. (2014) 42(4):161–8. 10.1249/JES.0000000000000023 25062002

[81] Thabane L Ma J Chu R Cheng J Ismaila A Rios LP A tutorial on pilot studies: the what, why and how. BMC Med Res Methodol. (2010) 10(1):1. 10.1186/1471-2288-10-1 20053272 PMC2824145

[82] Thabane L Cambon L Potvin L Pommier J Kivits J Minary L Population health intervention research: what is the place for pilot studies? Trials. (2019) 20(1):309. 10.1186/s13063-019-3422-4 31146768 PMC6543677

[83] Rogan S Karstens S. Verwendung der Begriffe Machbarkeits-bzw. Pilotstudien. Physioscience. (2018) 14(1):1–2. 10.1055/s-0044-100527

